# Mycobacterium tuberculosis osteomyelitis in a patient with human immunodeficiency virus/acquired immunodeficiency syndrome (HIV/AIDS): a case report

**DOI:** 10.1186/1757-1626-3-67

**Published:** 2010-02-23

**Authors:** Supriya Mannepalli, Levonne Mitchell-Samon, Nilmarie Guzman, Manish Relan, Yvette S McCarter

**Affiliations:** 1 Infectious Diseases Division, University of Florida College of Medicine-Jacksonville, Florida, USA; 2 Disease Control Division, Duval County Health Department, Jacksonville, Florida, USA; 3 Department of Pathology, University of Florida College of Medicine, Jacksonville, Florida, USA

## Abstract

The incidence of tuberculosis is increasing in the United States. Extra-pulmonary involvement is more common in patients with HIV/AIDS. The diagnosis of Tuberculosis osteomyelitis requires a high degree of suspicion for accurate and timely diagnosis.

We present a case of a 49 year old Caucasian male with HIV/AIDS who presented with a four-month history of soft tissue swelling in the left proximal thigh unresponsive to various broad spectrum antibiotics who was eventually diagnosed with Mycobacterium tuberculosis osteomyelitis of the left proximal femur.

## Case report

A 49 year old Caucasian male with HIV/AIDS was admitted with a four-month history of soft tissue swelling in the left proximal thigh. He initially noticed a small red nodule on the skin over the left thigh which eventually increased in size and ruptured with yellowish-green drainage. He reported that he had visited the emergency room at that time and was diagnosed as having a left thigh abscess which was incised and drained. No cultures were obtained during that visit. He was seen in follow-up at the HIV clinic where cultures of the drainage were obtained and he was given a prescription for minocycline. He had resolution of the drainage but relapsed 1 month later with pain and drainage from the left hip while still taking minocycline. He presented to the hospital in a "desperate attempt to seek medical attention" secondary to the persistent pain, swelling, yellowish drainage and difficulty with ambulation requiring the use of crutches.

On review of symptoms, the patient reported severe pain of the left lower extremity and hip which had gradually worsened to a severity scale of 10 out of 10. He denied any trauma to the site, associated fevers, or chills. No other acute symptoms were reported by the patient.

Patient denied any significant past medical history except HIV which was initially diagnosed in 2000. His nadir CD4 was 247 cells/mm^3 ^and he was started on antiretroviral therapy in 2001. At his last clinic visit less than 3 months prior, he had an absolute CD4 count of 540 cells/uL and a HIV RNA viral load of < 50 copies on his current regimen of zidovudine/lamivudine and lopinavir/ritonavir He reported no known drug allergies. In reviewing the out-patient records, patient also had a history of a positive tuberculin skin test in 2002 for which he reportedly took 6 months of isoniazid and vitamin B6. The outpatient records also indicated that he had complaints of swelling and drainage from the left hip as far back as 2002. He had one bacterial culture growing *Stenotrophomonas spp. *for which he was treated with trimethoprim-sulfamethoxizole and ciprofloxacin with partial resolution of drainage. Subsequently he had multiple negative bacterial cultures from this drainage, and multiple courses of antibiotics between 2002 and 2003. Specimens for acid-fast bacilli were sent in 2002 for which the smears were negative but the sample was insufficient for AFB culture. Since 2003 and the time of this presentation he has had episodes of exacerbations and remissions of the current problem but lacked funding for further diagnostic work-up. He smoked one pack of cigarettes per day but denied any alcohol or recreational drug use. He denied any recent travel or exposure to any animals. The patient had been homeless for several years, but denied history of incarceration.

On evaluation the patient was thin and poorly nourished, but in no acute distress. The blood pressure was 110/50 mm Hg, pulse 74 beats per minute, temperature 97.9°F, and respirations 18 breaths per minute. In general, he appeared to be cachectic. His cardiovascular, gastrointestinal and respiratory system examination was normal. A 7 cm × 4 cm fluctuant, tender soft tissue swelling was present on the lateral aspect of the left proximal thigh. A sinus tract was present distal to the left greater trochanter with yellowish green drainage oozing from the sinus and it could be probed with a swab to a depth of approximately 5 cm. There was no erythema of the surrounding skin. Motor and sensory examinations were normal and there was no lymphadenopathy noted in the inguinal areas.

Routine laboratory data were normal, including complete blood count, renal and liver function tests. Erythrocyte sedimentation rate (ESR) was 255. Plain x-ray images of the left hip and femur were negative for any bone abnormality. Blood cultures were negative.

**Figure 1:**
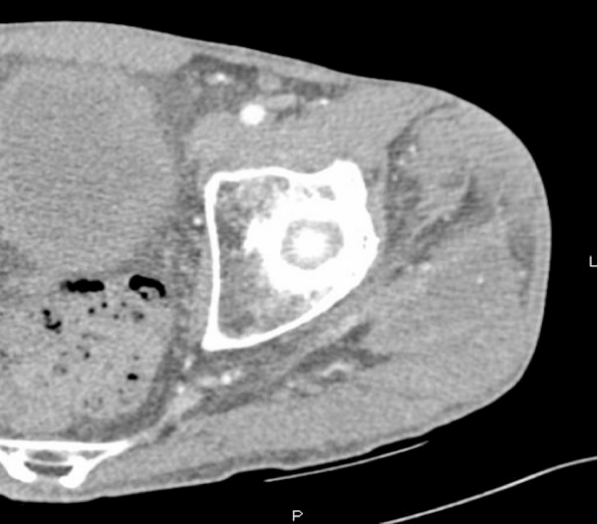
**CT scan of left lower extremity showing a 25 mm × 40 mm × 140 mm fluid collection (↔) and peripheral enhancement of the gluteus medius and vastus lateralis muscles**.

CT scan of the left lower extremity showed a 25 mm × 40 mm × 140 mm fluid collection with peripheral enhancement involving the gluteus medius and vastus lateralis muscles (Figure 1).

The patient was admitted with a diagnosis of left thigh soft tissue abscess with sinus tract. A bedside incision and drainage procedure was performed by General surgery. Cultures from the incision and drainage initially grew *Streptococcus agalactiae *and treatment was started with clindamycin IV by the primary team. Due to the chronicity of the wound, further evaluation with a MRI was pursued specifically to rule out underlying tumor or osteomyelitis.

**Figure 2:**
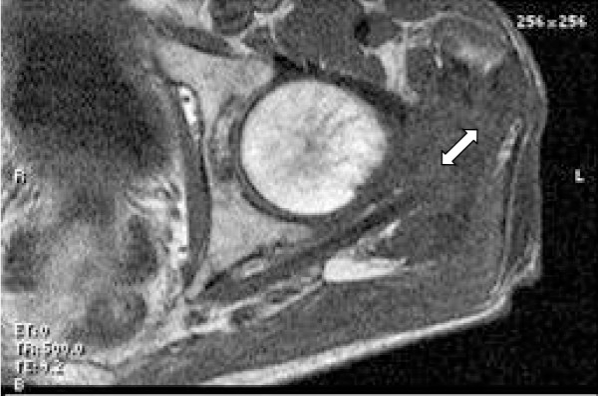
**MRI of the left thigh showing loculated fluid collection (↔) contiguous with the gluteus medius and pyriformis muscles**.

**Figure 3:**
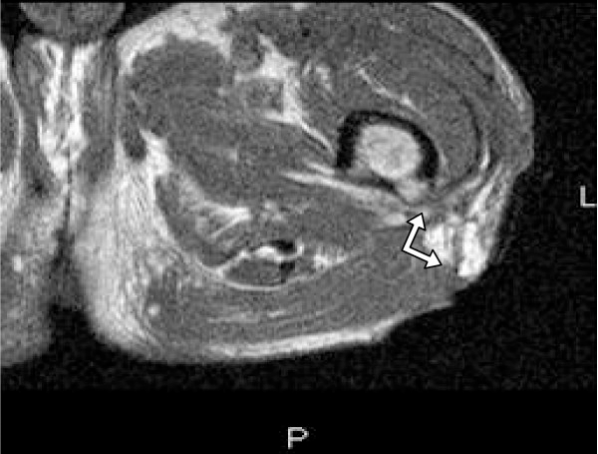
**MRI of the left thigh showing a sinus tract (↔) from the proximal femur**.

MRI of the left thigh showed a sinus tract within the proximal left thigh communicating with a loculated fluid collection contiguous with the gluteus medius and pyriformis muscles. It also revealed increased bone marrow signal intensity consistent with osteomyelitis of the proximal femur. (Figure 2 and 3)

The differential diagnosis considerations included chronic pyogenic osteomyelitis, primary bone tumor, secondary metastasis, inflammatory arthritis, sarcoma, or tuberculous osteomyelitis.

The antibiotics were changed at this time to meropenem, vancomycin and levofloxacin to empirically treat the most common etiologic agents of osteomyelitis while awaiting a more definitive diagnosis. Although tuberculous osteomyelitis was considered, it was lower on the differential and levofloxacin was added to give adequate therapy for pseudomonas as a potential pathogen. The patient then underwent a CT-guided percutaneous biopsy of the left greater trochanter to confirm the diagnosis. Cultures of the bone biopsy were negative for aerobic and anaerobic organisms as well as fungi. Acid fast bacilli smears were also negative. The histopathology on the CT-guided bone biopsy did not reveal any active osteomyelitis. The patient was transferred to our long-term care unit with the plan to complete a 6-week course of empiric treatment for osteomyelitis with piperacillin/tazobactam, vancomycin and levofloxacin. The cultures eventually grew *Mycobacterium tuberculosis *at 5 weeks.

The patient was then started on a 4-drug anti-tubercular therapy with isoniazid, rifampin, ethambutol, pyrazinamide and vitamin B6, based on the susceptibility pattern of the *Mycobacterium tuberculosis *isolated. A work-up to evaluate for pulmonary tuberculosis was negative. The patient was discharged with follow-up by a home wound care team, as well as, outpatient directly-observed therapy for tuberculous osteomyelitis for a total duration of nine months.

At his out-patient follow-up visit at our clinic he has shown good clinical improvement with complete closure of the sinus tract. He does, however, continue to complain of left hip pain but is able to ambulate without any assistive device. The Orthopedic service did not recommend any immediate surgical intervention and he will be followed up by orthopedics also as outpatient.

## Discussion

The incidence of tuberculosis is increasing in the United States [1]. Some of the factors attributing to this rise include the increasing number of people with HIV, increased international travel and the increase in the aging population (1). One fifth of the cases of tuberculosis have extra - pulmonary involvement and this is more common in patients with HIV/AIDS. HIV/AIDS is an important risk factor for reactivation of the latent tuberculous infection [2]. Due to the higher incidence of extra - pulmonary tuberculosis in HIV-infected patients, clinicians should always consider it in the differential diagnosis of osteomyelitis. An important opportunity for diagnosis was missed in this case at his emergency room visit when no cultures were sent from the incision and drainage procedure.

The diagnosis of tuberculous osteomyelitis requires a high degree of suspicion for accurate and timely diagnosis. Challenges in the diagnostic work up include lack of familiarity with the spectrum of tuberculous osteomyelitis bone lesions and not considering it in the differential early on [3]. The incidence of skeletal manifestations in tuberculosis is very low, (only 1-3%) and the spine is most commonly involved, followed by femur, tibia and fibula [4]. Tuberculous osteomyelitis occurs secondary to lymphohematogenous spread of *Mycobacterium tuberculosis from *a pulmonary focus [5].

Clinical symptoms are very nonspecific and can include insidious onset of pain, swelling, decreased range of motion and difficulty ambulating. Patients may also have weight loss, night sweats, generalized malaise and decreased appetite. Tuberculosis of the bone can go unnoticed for a long time until there is extension of the disease to skin and adjacent structures including the joints [[1,3], and [4]].

A significant challenge in the diagnosis of tuberculous osteomyelitis is that the smears for acid-fast bacilli are often negative, leading to a delay in diagnosis while waiting for the organisms to grow in culture media [6,7]. Polymerase chain reaction or nucleic amplification assays may be helpful in obtaining an earlier diagnosis; however, a negative result does not rule out tuberculosis.

Radiological findings include metaphyseal or epiphyseal destruction without sclerosis, sequestration, periosteal reaction and joint involvement. Unlike pyogenic osteomyelitis, there is sparing of the articular margins and cartilage space. Sometimes a solitary lytic lesion may be seen which can mimic neoplasia [[1,4], and [8]].

A positive tuberculin skin test is an important clue in patients with tuberculosis, but it can be negative in 10% of the patients. Although in some patients the ESR can be elevated, it is often normal [9,10]. The gold standard for diagnosis is the isolation of *Mycobacterium tuberculosis *from cultures of bone biopsy material. Histopathology of the bone lesions also is very helpful in confirming the diagnosis [[1,4], and [11]].

## Differential diagnosis

Differential diagnosis often includes chronic pyogenic osteomyelitis, primary bone tumor, secondary metastasis, granulomatous diseases, inflammatory arthritis and sarcoma [[4,6] and [12]].

## Treatment

Patients with a diagnosis of tuberculous osteomyelitis should be evaluated for pulmonary tuberculosis and in the hospital setting placing the patient in respiratory isolation would not be unreasonable, until this is ruled out. The Centers for Disease Control (CDC) published Guidelines for the Treatment of Opportunistic Infections recommends including a chest radiograph in the evaluation of suspected HIV-related TB regardless of the possible anatomic site of disease. "Sputum samples for AFB smear and culture should be obtained from patients with pulmonary symptoms and chest radiographic abnormalities. .....A positive AFB smear result in any specimen (sputum, needle aspirate, tissue biopsy) represents some form of mycobacterial disease but does not always represent TB. Because TB is the most virulent mycobacterial pathogen and can be spread from person to person, patients with smear-positive results should be considered to have TB disease until definitive mycobacterial species identification is made." [13]

Patients with the diagnosis of tuberculous osteomyelitis should be treated with anti-tubercular multi-drug therapy. First line treatment commonly includes isoniazid, vitamin B6, rifampin, ethambutol and pyrazinamide. Final therapy should always be based on the susceptibilities of the *Mycobacterium tuberculosis *isolated. Other second line agents include aminoglycosides, and quinolones [[1,6,11] and [14]].

The duration of treatment may vary from 6 months to 12 months. Patients may need up to 12 months or an even longer course of therapy based on the clinical response. The CDC recommends a 6- to 9-month regimen (2 months of INH, RIF, PZA, and EMB followed by 4--7 months of INH and RIF) for patients with extrapulmonary TB and states that 'for CNS disease and bone and joint TB, many experts recommend 9--12 months' [13] The World Health Organization (WHO) guidelines also endorse a 6-9 month regimen of anti-tuberculous therapy [14]. Surgery is rarely indicated in early cases. Radical debridement is only indicated in advanced disease, or if there is no improvement on medical therapy. Patients that develop significant joint involvement may need arthrodesis or total joint arthroplasty [[1,6,11], and [13]].

## Conclusion

Diagnosis of *Mycobacterium tuberculosis *osteomyelitis requires high degree of clinical suspicion for accurate and timely diagnosis. Tuberculous osteomyelitis should be considered in the differential in patients with persistent or recurrent skin and soft tissue infections, especially in patients with risk factors of immunosuppression or a positive tuberculin skin test.

## Consent

Written informed consent was obtained from the patient for publication of this case report and accompanying images. A copy of the written consent is available for review by the Editor-in-Chief of this journal.

## Competing interests

The authors declare that they have no competing interests.

## Authors' contributions

SM drafted the manuscript and participated in the management of this case. LM participated in the management of this case in the inpatient and outpatient setting and edited and revised the manuscript. NG participated in editing the manuscript. MR participated in the management of this case. YM participated in performing the laboratory diagnostic assays. All authors read and approved the final manuscript.
